# Hypokalemic Periodic Paralysis Precipitated by Thyrotoxicosis and Renal Tubular Acidosis

**DOI:** 10.1155/2021/4529009

**Published:** 2021-06-21

**Authors:** Ian Jackson, Yazan Addasi, Moeed Ahmed, Bashar Ramadan, Karson Kalian, Noor Addasi, Ali Nayfeh, Jocelyn Taylor, Khalid Bashir, Bryan Krajicek

**Affiliations:** ^1^Department of Medicine, Creighton University School of Medicine, Omaha, NE, USA; ^2^Department of Endocrinology, University of Nebraska Medical Center, Omaha, NE, USA; ^3^Department of Pulmonary and Critical Care, Creighton University School of Medicine, Omaha, NE, USA; ^4^Department of Nephrology, Creighton University School of Medicine, Omaha, NE, USA

## Abstract

**Background:**

Hypokalemic periodic paralysis is a rare neuromuscular disorder characterized by transient episodes of flaccid paralysis due to a defect in muscle ion channels. Most cases are hereditary, but it can be acquired. We present a case of acquired hypokalemic periodic paralysis associated with hyperthyroidism and renal tubular acidosis. *Clinical Case*. A 38-year-old female with a history of Graves' disease presented to the emergency department with generalized weakness and associated nausea, vomiting, and weight loss. Examination was significant for diffuse weakness in all extremities. Labs showed hypokalemia, hyperthyroidism, and nonanion gap metabolic acidosis with a positive urine anion gap. She was treated for hypokalemic periodic paralysis and renal tubular acidosis. Potassium replacement, propranolol, methimazole, and sodium bicarbonate were initiated. Her potassium gradually corrected with resolution of her symptoms. Further investigation revealed a history of dry eyes, dry mouth, and recurrent dental carries. She had positive ANA, SS-A, and SS-B antibodies. She was diagnosed with Sjögren's syndrome, which may have been associated with her Graves' disease and thus contributed to both her RTA and hyperthyroidism.

**Conclusion:**

Early recognition and treatment of thyrotoxic periodic paralysis are important to prevent cardiac complications. Management includes potassium replacement with careful monitoring to prevent rebound hyperkalemia. The definitive treatment is to achieve euthyroid status.

## 1. Introduction

Periodic paralysis is a rare neuromuscular disorder caused by a defect in muscle ion channels. It is characterized by episodes of painless muscle weakness. Hypokalemic periodic paralysis (HPP) is a subset of periodic paralysis that occurs in the presence of low serum potassium. HPP may be inherited or occur sporadically in the setting of hyperthyroidism. We are presenting a rare case of HPP associated with hyperthyroidism and renal tubular acidosis. We discuss the pathophysiology, clinical manifestations, assessment, and management of HPP.

## 2. Case Presentation

A 38-year-old female with a medical history of Graves' thyrotoxicosis presented to the emergency department (ED) complaining of generalized weakness that started several days prior to her presentation. It was associated with nausea, vomiting, and weight loss. She denied diarrhea, pain, or shortness of breath. Her only medication was methimazole 10 milligrams twice daily which she had been taking for three years with intermittent compliance. She was alert and oriented to person, place, and time. Her vitals on presentation were blood pressure of 127/80 mmHg, heart rate of 80, respiratory rate of 22, and temperature of 98.4°F. Neurological exam yielded intact cranial nerves with no sensory deficit. However, she had diffuse weakness in all extremities with strength of 2/5 in the lower extremities and 2/5 in the upper extremities. The rest of the physical examination showed no abnormalities.

**Figure 1:**
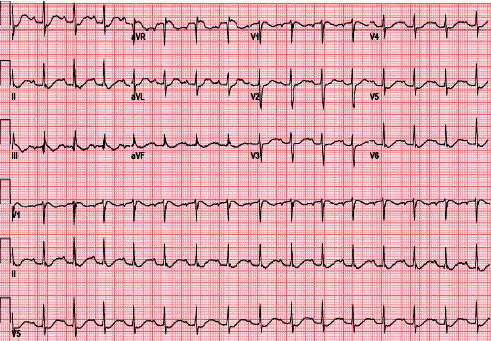
EKG with changes indicative of hypokalemia.

Workup in the ED revealed multiple lab abnormalities including hypokalemia, a nonanion gap metabolic acidosis, and hyperthyroidism (Table 1). Her EKG showed changes consistent with hypokalemia including a QTc interval of 686 milliseconds (Figure 1). A chest X-ray and computed tomography of the head without contrast showed no acute abnormalities. Renal ultrasound and abdominal X-ray were negative for hydronephrosis or kidney stones.

The patient was given intravenous potassium in the ED and transferred to the intensive care unit (ICU) with continuous cardiac monitoring. The endocrinology team recommended starting propranolol and increasing her dose of methimazole. She was treated with additional intravenous and oral potassium supplementation in the ICU. The potassium level was checked frequently due to concern for rebound hyperkalemia. Based on the patient's acute onset of diffuse weakness without cranial nerve dysfunction or pain, hypokalemia, elevated free T4, and low TSH, a presumptive diagnosis of thyrotoxic periodic paralysis was made.

**Figure 2:**
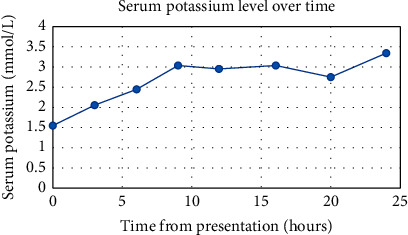
Serum potassium level over time.

The patient's potassium was gradually corrected with subsequent resolution of her paralysis and decrease of her QTc interval. However, she continued to have a nonanion gap acidosis. Urine studies showed a urine pH of 8 (normal range 4.5–8), urine chloride of 105 mmol/L (normal range 55–125 mmol/L), urine potassium of 39.8 mmol/L (normal range 12–62 mmol/L), and urine sodium of 90 mmol/L (normal range 20–110 mmol/L) resulting in a positive urine anion gap of 24.8. This was attributed to renal tubular acidosis (RTA) type 1. The patient was started on sodium bicarbonate in addition to her oral potassium supplementation. These medications were gradually titrated and later discontinued as her labs normalized (Figure 2).

Further history revealed intermittent dry eyes, dry mouth, and recurrent dental carries. This prompted additional workup which was significant for positive ANA, SS-A, and SS-B. The patient was eventually referred to rheumatology for further evaluation and management of Sjögren's syndrome. She was started on hydroxychloroquine. With this treatment, her RTA completely resolved, and she no longer required potassium or bicarbonate supplementation. The patient maintained follow-up with endocrinology, and she preferred to continue antithyroid medications rather than pursue radioactive iodine ablation.

## 3. Discussion

Periodic paralysis is a neuromuscular disorder associated with defective muscle ion channels and characterized by episodes of painless muscle weakness. It is classified as hypokalemic or hyperkalemic based on the serum potassium level. The majority of cases are familial and inherited in an autosomal dominant pattern. Less commonly, hypokalemic periodic paralysis (HPP) can also be acquired in patients with thyrotoxicosis. Thyrotoxic periodic paralysis (TPP) can be caused by any form of thyrotoxicosis but is most common in Graves' disease. Unlike other forms of thyroid disease, TPP is more common in males. It is also more frequent in Asian populations with an incidence of approximately 2% in patients with thyrotoxicosis [1].

Although the exact pathogenesis is unclear, it is hypothesized that patient's with TPP have an underlying defect in muscle ion channels that remains asymptomatic in patients with normal thyroid function. However, when thyroid hormone levels are elevated, it increases tissue responsiveness to beta-adrenergic stimulation. This in turn increases sodium-potassium ATPase activity and drives potassium into cells. The intracellular shift of potassium causes hyperpolarization of the muscle membrane, resulting in inexcitability of the muscle. It is not caused by a deficiency in total body potassium [2]. Furthermore, obese patients are more susceptible to episodes of TPP because high levels of insulin also stimulate a shift of potassium into cells [3, 4].

TPP manifests as transient episodes of painless muscle weakness with preserved consciousness. The episodes typically last for hours to days. The weakness is generalized, but often more pronounced in proximal muscles. Episodes can be exacerbated by high carbohydrate foods, exercise, stress, infection, anesthesia, or exposure to extreme temperatures [5]. Our patient reported having a large fast-food meal the night prior to onset of her symptoms. Other less common symptoms include myalgia, hyporeflexia or areflexia, and associated hyperthyroid symptoms such as tachycardia. TPP can rarely cause potentially fatal arrhythmias.

Diagnosis of TPP should be suspected when a patient presents with paralysis associated with hypokalemia and hyperthyroidism. TPP must be distinguished from other causes of acute paralysis including myasthenic crisis, botulism, Guillain–Barre syndrome, acute myelopathy, and acute thyrotoxic myopathy. In our case, the patient had normal cranial nerve function, making myasthenic crisis or botulism unlikely. Her weakness was present diffusely without an ascending pattern that would indicate Guillain–Barre syndrome. Her lack of pain, normal creatine kinase level, and nondermatomal distribution were also inconsistent with acute thyrotoxic myopathy or acute myelopathy. Paralysis in TPP usually occurs when the potassium level is less than 3 mmol/L. In one study of 78 patients with TPP, the mean serum potassium level was 2.1 mmol/L [6]. EKG changes often include tachycardia and changes consistent with hypokalemia.

Acute treatment of TPP primarily involves potassium supplementation. Oral potassium chloride is the preferred method, but intravenous potassium may be necessary in patients with impaired swallowing. One protocol suggests a dose of 30 milliequivalents orally every 15–30 minutes until serum potassium is normalized [7]. Rebound hyperkalemia is common, so close monitoring of serum potassium and cardiac telemetry should be used. In cases refractory to potassium replacement, intravenous propranolol may help to reverse the excess beta-adrenergic stimulation causing potassium to shift into the cells. Long-term preventative treatment of TPP is through restoration of euthyroid state, which can be accomplished through antithyroid medications, radioactive iodine ablation, or thyroidectomy.

RTA secondary to Sjögren's syndrome may have also contributed to HPP in this case. Approximately 5% of patients with primary Sjögren's syndrome have clinically significant renal involvement, with distal RTA being the most common manifestation. The resulting hypokalemia and metabolic acidosis can contribute to muscle weakness and periodic paralysis [8]. Corticosteroids are the most widely used therapy. The efficacy of steroid-sparing agents, such as hydroxychloroquine used for our patient, remains unknown [9].

Our patient had several atypical features for HPP. First, acquired cases of HPP are far more common in Asian males, while our patient was a Caucasian female. Second, the presence of both thyrotoxicosis and renal tubular acidosis as underlying causes of HPP is very rare. The combination of these two mechanisms in our patient is likely due to her diagnosis of Sjögren's syndrome, which can not only cause distal RTA but has also been associated with autoimmune thyroid disorders. The coexistence of Sjögren's syndrome and autoimmune thyroid disorders, such as Graves' disease in our patient, is potentially due to common pathogenetic mechanisms shared by the two conditions [10].

## 4. Conclusion

Early diagnosis and treatment of TPP are important to prevent serious cardiac complications. Potassium replacement should be gradual, with serial monitoring of potassium to avoid rebound hyperkalemia. The definitive treatment is to achieve euthyroid status to prevent recurrent attacks.

## Figures and Tables

**Table 1:** Initial laboratory values.

Lab	Result	Normal range
Sodium	142 mmol/L	135–144 mmol/L
Potassium	1.6 mmol/L	3.7–5.1 mmol/L
Chloride	114 mmol/L	96–110 mmol/L
CO_2_	16 mmol/L	22–32 mmol/L
Creatinine	0.92 mg/dL	0.5–1.6 mg/dL
Magnesium	2.3 mg/dL	1.8–2.6 mg/dL
Phosphorus	1.4 mg/dL	2.5–4.9 mg/dL
pH	7.25	7.35–7.45
pCO_2_	34 mmol/L	35–45 mmol/L
TSH	<0.005 UIU/mL	0.4–3.8 UIU/mL
T4, free	1.6 ng/dL	0.7–1.4 ng/dL

## Data Availability

No additional data were accessed or generated for this study.
